# Nomogram for Estimating the Risks of Intestinal Ischemia and Necrosis in Neonates With Midgut Volvulus: A Retrospective Study

**DOI:** 10.3389/fped.2022.888594

**Published:** 2022-06-20

**Authors:** Xisi Guan, Zhe Wang, Qiuming He, Junjian Lv, Jiakang Yu, Wei Zhong

**Affiliations:** Department of Neonatal Surgery, Guangzhou Women and Children’s Medical Center, Guangzhou, China

**Keywords:** midgut volvulus, neonate, intestinal ischemia and necrosis, nomogram, risk factors

## Abstract

**Background:**

Delayed diagnosis and inaccurate judgment of the severity of the disease may be the principal reasons for the poor prognosis associated with neonatal midgut volvulus. We aimed to develop a nomogram model that timely assesses the risks of intestinal ischemia and necrosis in the neonate with midgut volvulus.

**Materials and Methods:**

We retrospectively analyzed the clinical data from neonates with midgut volvulus who were admitted to Guangzhou Women and Children’s Medical Center from January 2009 to December 2019. Univariate and multivariate analyses were used to obtain independent factors to build a predictive model. The independent factors were used to develop the nomogram model.

**Results:**

Heart rate, mean arterial pressure, serum C-reactive protein, serum sodium, serum albumin, and pH levels were independent predictors for intestinal ischemia and necrosis in patients with midgut volvulus. The area under the receiver operating characteristic curve (AUC) of the predictive model was 0.985 (95% confidence interval, 0.966–0.999; *P* < 0.001). The sensitivity was 90.48%, and the specificity was 93.10%. A nomogram model was established using the six independent predictors, with a C-index of 0.859 and a favorable consistency between the predicted and actual intestinal ischemia and necrosis rates according to the internal validation.

**Conclusion:**

The constructed nomogram model could be a superior tool for predicting intestinal ischemia and necrosis in neonates with midgut volvulus.

## Background

Midgut volvulus (MGV) refers to a condition in which the midgut twists around the axis of the superior mesenteric artery and is often related to malrotation (1). The incidence of MGV in the general population ranges between 1.7 and 60 per 100,000 individuals (2). MGV is most likely to occur in neonates and young infants. Compared to intestinal necrosis caused by other common diseases in the neonatal period, intestinal necrosis caused by MGV has its uniqueness. For example, unlike neonatal necrotizing enterocolitis, it is not caused by inflammation but by intestinal ischemia and infarction. Furthermore, compared with segmental volvulus and internal hernia, the consequences of MGV are more serious as it can lead to irreversible intestinal necrosis, and extensive resection may be necessary. It has also been reported that acute MGV is the most severe form of volvulus and carries a fourfold risk of mortality compared with segmental volvulus (3), with an overall mortality rate of 2.2–16% (4–6). Therefore, we may need more timely and objective indicators to help clinicians judge whether neonates with MGV have intestinal ischemia and necrosis so as to expedite the operation and thereby save more neonates’ intestines. However, few researchers have focused on this important question.

In this study, we aimed to develop a nomogram model based on objective clinical data available within 2 h of admission to predict whether neonates with MGV have intestinal ischemia and necrosis. This new model is expected to help clinicians accurately estimate the urgency of treating neonates with MGV and perform super emergency operations to improve the prognosis.

## Patients and Methods

Neonates with a preoperative suspected diagnosis of MGV admitted to Guangzhou Women and Children’s Medical Center from January 2009 to December 2019 were enrolled in the present study. The preoperative suspected diagnosis was based on medical history, physical examination, ultrasound, or upper gastrointestinal radiography. The final diagnosis of MGV and bowel ischemia and necrosis was determined based upon the operative and pathologic findings. Patients with a surgically confirmed absence of midgut volvulus were excluded. Cases with severe cardiac malformations, congenital diaphragmatic hernia, gastroschisis, omphalocele, and other digestive tract malformations were also excluded.

Patients were apportioned to an intestinal ischemia and necrosis group (*n* = 21) or a non-intestinal ischemia and necrosis group (*n* = 87), with the latter population randomly selected from among 210 cases without intestinal ischemia and necrosis.

We retrospectively analyzed the objective clinical data available within 2 h of admission. The selection of these variables was based on the published literature (5, 7–11). The variables of interest included personal history, age at onset, first symptom observed, vital signs measured immediately after admission, laboratory variables determined within 2 h after admission, X-ray evaluated within 2 h after admission, and days from symptom to operation.

Univariate and multivariate analyses were used to obtain independent factors to build a predictive model. Leave-one-out cross-validation (LOO-CV) was employed for the internal validation of the predictive model. The independent factors were used to develop the nomogram model. The predictive accuracy of the nomogram was determined by concordance index (C-index) calculation and calibration plots.

The present study was approved by the ethics committee of Guangzhou Women and Children’s Medical Center (no. 192A01), and written informed patient consent was obtained from each patient’s family.

### Statistical Methods

**FIGURE 1:**
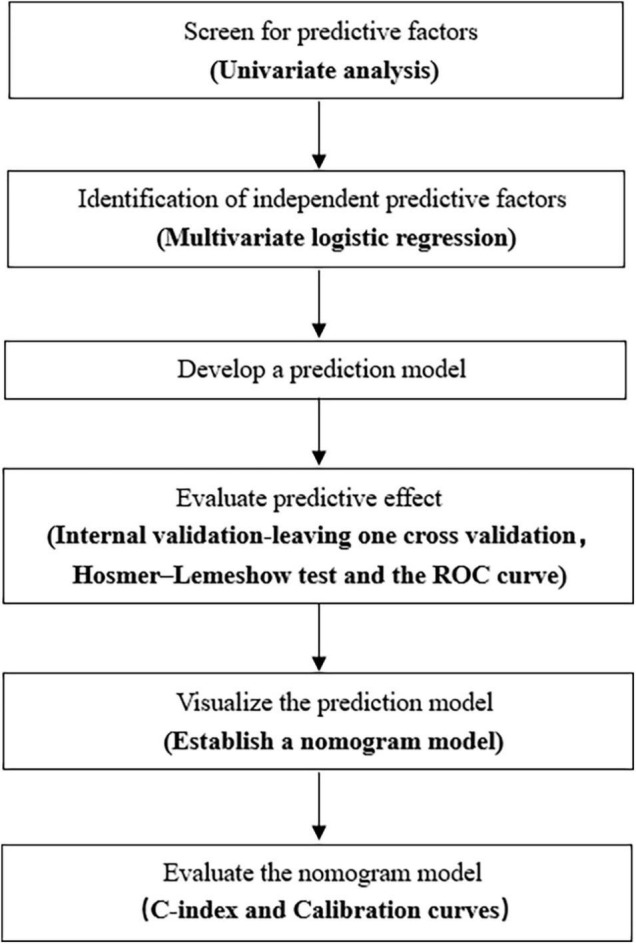
A flowchart displaying the process of building the nomogram.

We performed statistical analysis using SAS version 9.4 (SAS Institute, Cary, NC, United States). Categorical variables were expressed as numbers and percentages and were compared using the chi-squared test and Fisher’s exact probability test. Measurement data that were not normally distributed are represented by median (interquartile range) values. Comparisons between two groups were performed by the Wilcoxon rank-sum test. Meanwhile, those data conforming to normal distribution were described by mean ± standard deviation values, and comparisons between the two groups were performed by *t*-test. Variables showing statistical significance (*P* < 0.05) in the univariate analysis were brought into the logistic regression and further screened by stepwise regression. Hosmer-lemeshow test and receiver operating characteristic (ROC) curve were used to evaluate the prediction model. An internal validation of the prediction model was carried out by leaving one cross-validation. The nomogram prediction model was established using the RMS package of R version 3.6.1 (R Foundation for Statistical Computing, Vienna, Austria). The nomogram was internally validated using bootstraps with 2,000 resamples. We used the Harrell concordance index (C-index) in the nomogram to assess the model performance, and a two-sided *P* value < 0.05 was considered to be statistically significant. A flowchart displaying the process of building the nomogram is shown in Figure 1.

## Results

### Identification of Predictive Factors for Intestinal Ischemia and Necrosis Caused by Midgut Volvulus

(1) Intestinal ischemia and necrosis during the operation were found in 21 of the final 231 cases (9.1%). There was no significant difference in sex, gestational age, delivery method, or birth weight between the two groups (*P* > 0.05; Table 1). The median time from admission to surgery was 6 h (4–8 h). Among the 21 children with intestinal ischemia and necrosis, 7 had total intestinal necrosis, and the remaining small intestine length was less than 30 cm in 6 cases, 31–60 cm in 2 cases, 61–90 cm in 3 cases, and >90 cm in 3 cases. In the 3 cases with the remaining intestine >90 cm, 2 cases were confirmed by the first operation for severe intestinal ischemia, a large area of mesenteric thrombosis, but no necrosis, and, in the second surgery, we found that all intestinal blood supply was restored.

**TABLE 1:** Analyses of clinical and laboratory parameters in midgut volvulus.

Variables	Intestinal necrosis (*n* = 21)	Non-intestinal necrosis (*n* = 87)	*P*
**Features**			
Male sex, *n* (%)	13 (61.90%)	67 (77.01%)	0.156
Preterm births (< 37 weeks), *n* (%)	4 (19.05%)	5 (5.75%)	0.07
Cesarean sections, *n* (%)	8 (38.10%)	23 (26.44%)	0.289
Gestational weight (kg)*	2.96 (2.45,3.16)	3.03 (2.8,3.35)	0.050
Age at admission (d)*	4 (2,6)	8 (5,11)	0.003
Age at onset (d)*	1 (1,4)	2 (1,5)	0.182
**First symptoms, *n* (%)**			
Bilious vomiting	15 (71.43%)	85 (97.70%)	**0.001**
Hematochezia	2 (9.52%)	2 (2.30%)	0.169
Abdominal distention	2 (9.52%)	0 (0.00%)	**0.036**
**Vital signs**			
Heart rate*	153 (140,164)	140 (130,146)	**0.005**
MAP (mmHg)*	62 (58.67,70.67)	54 (50.67,60.33)	**0.001**
RR*	45 (41,48)	44 (40,46)	0.346
**Laboratory findings**			
Lactic acid (mmol/L)*	4.00 (2.80,4.70)	3.50 (2.50,4.90)	0.41
HGB (g/L)**	115.71 ± 30.21	146.79 ± 25.37	**<0.001**
Leukocyte (10^9^/L)*	15.80 (10.60,20.70)	10.70 (8.80,13.50)	**0.009**
CRP (mg/L)*	57.05 (10,84.80)	1.34 (0.51,4.59)	**<0.001**
Glucose (mmol/L)*	6.00 (5.20,7.30)	4.87 (4.12,5.90)	**0.006**
Albumin (g/L)*	34.70 (31.30,36.20)	38.80 (35.90,42.10)	**<0.001**
K(mmol/L)**	4.80 ± 0.80	3.92 ± 0.54	**<0.001**
Na (mmol/L)**	134.20 ± 3.40	136.53 ± 4.19	**0.02**
PH**	7.37 ± 0.11	7.46 ± 0.07	**0.001**
BE (mmol/L)**	−4.14 ± 5.21	−1.05 ± 4.34	**0.006**
**X-ray (pneumoperitoneu m), *n* (%)**	2 (9.52%)	0 (0.00%)	**0.036**
**Other**			
Days from symptoms to operation (d)*	1.00 (0.75,3.00)	7.00 (4.00,12.00)	**<0.001**

*Median (P25, P75) ** Mean (SD). BE, base excess; CRP, C-reactive protein; HGB, Hemoglobin; MAP, mean arterial pressure; RR, respiratory rate. Significant p values (p < 0.05) are bolded.

(2) According to univariate analysis, the median age at admission and the incidence of biliary vomiting in the intestinal ischemia and necrosis group were significantly lower than in the non-intestinal ischemia and necrosis group (97.7%; *P* < 0.01); the incidences of abdominal distension, heart rate (HR), and mean arterial pressure measured within 2 h after admission in the former group were significantly higher than those in the latter group (0%; *P* < 0.05). In the intestinal ischemia and necrosis group, the levels of white leukocytes, C-reactive protein (CRP), glucose, and blood potassium detected within 2 h of admission were significantly higher than those in the non-intestinal ischemia and necrosis group, while the levels of hemoglobin, blood sodium, serum albumin, pH, and base excess (BE) were significantly lower than those in the non-intestinal ischemia and necrosis group (*P* < 0.05).

(3) In the multivariate analysis, HR, mean arterial pressure (MAP), CRP, serum sodium, albumin (ALB), and pH value determined within 2 h after admission were the independent predictors of intestinal ischemia and necrosis in patients with MGV (Table 2).

**TABLE 2:** Multivariate logistic regression model of intestinal necrosis.

Variables	B	SE	*P*	OR (95%CI)
Intercept	132.79	54.389	0.015	–
HR, time/min	0.116	0.054	0.032	1.123 (1.010–1.249)
MAP, mmHg	0.116	0.059	0.050	1.123 (1.000–1.261)
CRP, mg/L	0.076	0.025	0.003	1.079 (1.027–1.134)
Na, mmol/L	–0.435	0.199	0.029	0.647 (0.438–0.956)
ALB, g/L	–0.342	0.14	0.014	0.710 (0.540–0.933)
pH value × 10^–1^	–1.317	0.636	0.038	0.268 (0.077–0.931)

*ALB, albumin; CI, confidence interval; CRP, C-reactive protein; MAP, mean arterial pressure; OR, odds ratio.*

### Development and Internal Validation of the Prediction Model

The prediction model formula established was as follows:


$$P=\frac{e^{\begin{array}{c} (132.79+0.116\times \text{HR}+0.116\times \text{MAP}+0.076\times \text{CRP}\\ -0.435\times \text{Na}-0.342\times \text{ALB}-1.317\times \mathrm{pH}\mathrm{value}) \end{array}}}{1+e^{\begin{array}{c} (132.79+0.116\times \text{HR}+0.116\times \text{MAP}+0.076\times \text{CRP}\\ -0.435\times \text{Na}-0.342\times \text{ALB}-1.317\times \mathrm{pH}\mathrm{value}) \end{array}}}$$

**FIGURE 2:**
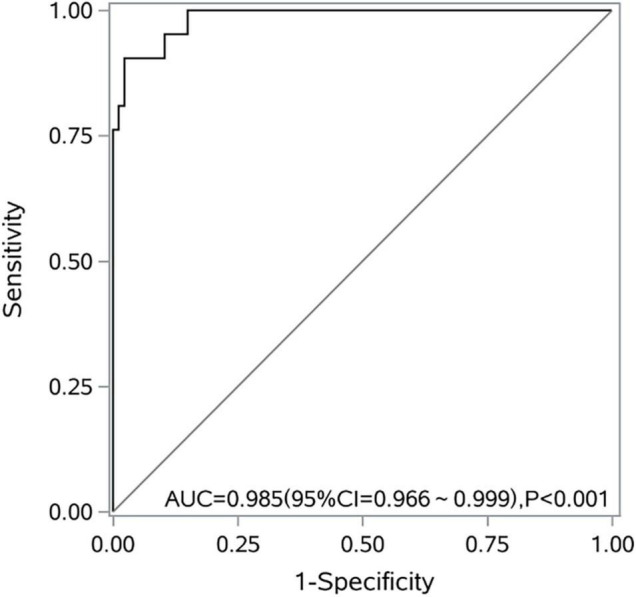
A receiver operating characteristic curve of the logistic regression model for predicting intestinal ischemia and necrosis caused by midgut volvulus.

The model showed a good fit (Hosmer–Lemeshow test χ2 = 0.969; *P* = 0.998; max-rescaled *R*^2^ = 0.837). The area under the ROC curve was 0.985 (95% CI, 0.966–0.999; *P* < 0.001). When *P* > 0.2 (cut-off value), it was judged as intestinal ischemia and necrosis. Leave-one-out cross-validation (LOO-CV) was employed for the internal validation of the predictive model. The accuracy was 92.59%, the sensitivity was 90.48%, the specificity was 93.10%, the positive predictive value was 76.00%, and the negative predictive value was 97.59% (Figure 2).

### Development and Internal Validation of the Nomogram Model

**FIGURE 3:**
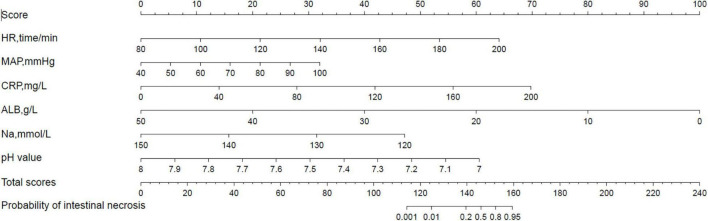
A nomogram model for predicting intestinal ischemia and necrosis in neonates with midgut volvulus.

**FIGURE 4:**
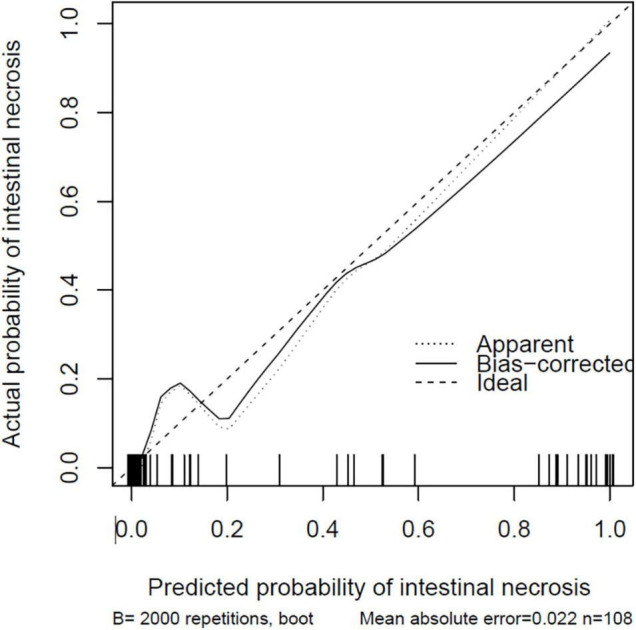
The correction curve for the nomogram model.

The nomogram model is constructed according to the factors in the multivariate logistic regression model (Figure 3). The total score is obtained by adding each index. The probability of intestinal ischemia and necrosis can be obtained according to the nomogram. When the total score exceeds 140 points (*P* = 0.20), it is considered indicative that the patient has intestinal ischemia and necrosis. After 2,000 bootstrap self-help sampling verifications, the nomogram model predicted intestinal ischemia and necrosis with a C-index of 0.859 and a corrected C-index of 0.855, with good discrimination. The correction curve is shown in Figure 4. The intestinal ischemia and necrosis probability obtained by the model is in good agreement with the actual observation, with an average absolute error of 0.022.

### Clinical Application

A high index of suspicion for midgut volvulus is based on the history and physical examination findings. In neonates, MGV typically presents with the sudden onset of bilious vomiting, and soon after the onset of vomiting, the lower abdomen may appear scaphoid. With complete obstruction, the patient develops intestinal ischemia with abdominal distension, bloody stools, and lethargy. Doppler ultrasonography or upper GI contrast radiography can assist doctors in the diagnosis. If the diagnosis of midgut volvulus is highly suspected, the nomogram model can be used to identify whether intestinal ischemia or intestinal necrosis is present.

For example, if a patient highly suspected of having midgut volvulus has an HR of 120 beats/min, a score of 21 points is assigned; for a MAP of 70 mmHg, a score of 16 points is assigned; for a CRP level of 75 mg/L, a score of 26 points is assigned; for an Na concentration of 135 mmol/L, a score of 24 points is assigned; for an ALB concentration of 40 g/L, a score of 20 points is assigned; for a pH value of 7.42, a score of 35 points is assigned. Then, the total score is 142 points, and the probability of intestinal ischemia and necrosis is 0.3; thus, it is judged that the patient has intestinal ischemia and necrosis, and we should immediately give the patient absolute priority for surgery.

## Discussion

MGV is often caused by congenital intestinal malrotation, a variation in intestinal position, and incomplete mesenteric attachment. Approximately 75% of MGV cases occur in the first year of life, and most cases occur during the neonatal period (12, 13).

The prognosis of patients with MGV displays a polarizing trend, with children exhibiting a good prognosis achieving a quality of life similar to that of the normal population, while children exhibiting a poor prognosis suffer serious complications, such as short bowel, total intestinal necrosis, and even death (14). The incidence of bowel necrosis with MGV in other reports was found to range from 2.9 to 48% (7, 15). In our cases, the median time from admission to surgery was 6 h, and the probability of intestinal necrosis was 9.1%, which is not a satisfactory result. However, in our study, 2 cases were confirmed by the first operation for severe intestinal ischemia, a large area of mesenteric thrombosis, but no necrosis. Then, during the second surgery, we found that all intestinal blood supply was restored, with a median time from admission to surgery of 4 h recorded among cases, which suggests that timely surgical intervention may change the prognosis.

In our study, based on univariate and multivariate logistic regression analyses, it was concluded that HR; MAP; pH; and serum CRP, sodium, and albumin levels are independent predictors for intestinal ischemia and necrosis in patients with MGV. Using these predictors, we developed a nomogram to visualize the results of the regression analysis; when the total score exceeds 140 points (P = 0.20), it is considered indicative of the patient having intestinal ischemia and necrosis.

Intestinal necrosis often appears as dehydration and shock, and the early manifestations of shock are increased HR and increased blood pressure. Silvia et al. reported that the major risk factor for death among neonates with an intestinal volvulus is hemodynamic instability (3). A strangulated small intestine can lead to increased vascular permeability and intestinal bacterial translocation, which can then also cause changes in blood inflammatory indicators. In multivariate analyses from other studies (8, 9), elevated leukocyte count and CRP levels were independent risk factors for intestinal gangrene caused by volvulus. Similarly, in a study by Chang (10), abdominal pain, abdominal distension, tachycardia, and elevated white blood cell count were independent risk factors for intestinal necrosis caused by strangulated intestinal obstruction. Meanwhile, Li (16) found that CRP level was able to reflect the inflammatory condition of strangulated intestinal obstruction with necrosis and showed a good ability to distinguish between strangulated intestinal obstruction and simple intestinal obstruction. Hyponatremia has previously been identified as a predictor of the presence of ischemia in a variety of surgical emergencies, including necrotizing soft tissue infections, gangrenous cholecystitis, ischemic bowel in children with volvulus, and ischemic bowel in the setting of small-bowel obstruction (8, 11). Lin et al. (8) found that hyponatremia (<130 mmol/L) is significantly associated with bowel gangrene in small-bowel volvulus. In addition, previous literature reports that metabolic acidosis was present in 75% of those with strangulated bowel (8). Ilias et al. (5) suggested that the results of immediate blood gas analysis were better able than inflammatory indicators to predict intestinal necrosis caused by neonatal volvulus; they also found that the pH and BE values for children with complex volvulus were significantly reduced (*P* < 0.05).

In addition, we newly uncovered serum albumin – a negative acute-phase reactant synthesized by the liver – as an independent risk factor for intestinal necrosis caused by MGV. However, in the presence of systemic inflammation, the liver synthesizes acute-phase proteins other than serum albumin. Therefore, some people posit that low serum albumin levels reflect an inflammatory state (17). However, the correlation between serum albumin and inflammatory markers such as CRP, interleukin-6, and tumor necrosis factor remains unclear, and other pathways apart from inflammatory ones may be involved (18). We uncovered no studies in the literature commenting on the study by Lee (19), who found that the total protein in their intestinal necrosis group was significantly lower than that of the non-intestinal necrosis group when they analyzed infant intestinal malrotation and catastrophic volvulus (*P* < 0.05). We plan to further verify some of our novel findings in subsequent studies.

There were several limitations of our current study. As this study was a preliminary and single-center retrospective study and the number of cases included was relatively limited, the findings need to be externally evaluated in a greater number of neonates with MGV. In the future, a larger prospective study involving multiple centers and a larger number of patients should be undertaken to validate and improve the nomogram.

## Conclusion

HR, MAP, serum CRP, albumin, serum sodium levels, and pH values were the factors closely related to intestinal ischemia and necrosis. The constructed nomogram model, which combines the HR, MAP, serum CRP, albumin, serum sodium levels, and pH values, can be a superior tool for predicting intestinal ischemia and necrosis in neonates with midgut volvulus.

## Data Availability Statement

The original contributions presented in this study are included in the article/supplementary material, further inquiries can be directed to the corresponding author.

## Ethics Statement

The studies involving human participants were reviewed and approved by Guangzhou Women and Children’s Hostipal. Written informed consent to participate in this study was provided by the participants’ legal guardian/next of kin.

## Author Contributions

XG, ZW, and WZ: study concept and design. XG: drafting of the manuscript, statistical analysis, and interpretation of data. WZ, ZW, and QH: critical revision of the manuscript for important intellectual content. JY and JL: administrative, technical, or material support. All authors contributed to the manuscript revision, read, and approved the submitted version.

## Conflict of Interest

The authors declare that the research was conducted in the absence of any commercial or financial relationships that could be construed as a potential conflict of interest.

## Publisher’s Note

All claims expressed in this article are solely those of the authors and do not necessarily represent those of their affiliated organizations, or those of the publisher, the editors and the reviewers. Any product that may be evaluated in this article, or claim that may be made by its manufacturer, is not guaranteed or endorsed by the publisher.
